# Barium/Cobalt@Polyethylene Glycol Nanocomposites for Dye Removal from Aqueous Solutions

**DOI:** 10.3390/polym13071161

**Published:** 2021-04-05

**Authors:** Somayeh Rahdar, Abbas Rahdar, Mostafa Sattari, Laleh Divband Hafshejani, Athanasia K. Tolkou, George Z. Kyzas

**Affiliations:** 1 Department of Environmental Health, Zabol University of Medical Sciences, Zabol 9861615881, Iran; rahdar89@gmail.com; 2 Department of Physics, Faculty of Science, University of Zabol, Zabol 538-98615, Iran; 3 Department of Mathematics, Faculty of Science, University of Zabol, Zabol 538-98615, Iran; msattari.b@gmail.com; 4 Department of Environmental Engineering, Faculty of Water and Environmental Engineering, Shahid Chamran University of Ahvaz, Ahvaz 6135743136, Iran; l.divband@scu.ac.ir; 5 Department of Chemistry, Aristotle University of Thessaloniki, 54124 Thessaloniki, Greece; tolkatha@chem.auth.gr; 6 Department of Chemistry, International Hellenic University, 65404 Kavala, Greece

**Keywords:** dyes, acid blue 92, nanocomposite, adsorption, isotherm, kinetics

## Abstract

Dyes are known as one of the most dangerous industrial pollutants which can cause skin diseases, allergy, and provoke cancer and mutation in humans. Therefore, one of the important environmental issues is the effective removal of dyes from industrial wastewater. In the current work, BaFe_12_O_19_/CoFe_2_O_4_@polyethylene glycol (abbreviated as BFO/CFO@PEG) nanocomposite was synthesized and evaluated regarding its capacity for adsorptive removal of a model dye Acid Blue 92 (denoted as AB92) from aqueous solutions. The characteristics of the prepared nanocomposite was determined by tests such as X-ray diffraction (XRD), scanning electron microscope (SEM), vibration sample magnetization (VSM), and Fourier transform infrared spectroscopy (FTIR). The effects of conditional parameters including pH (2–12), initial concentration of dye (20–100 mg/L), adsorbent dosage (0.02–0.1 g/L) and contact time (0-180 min) on the adsorption of dye were investigated and then optimized. The results indicated that with the increase of the adsorbent dosage from 0.02 to 0.1 g/L, the removal efficiency increased from 74.1% to 78.6%, and the adsorbed amount decreased from 148.25 to 31.44 mg/g. The maximum removal efficiency (77.54%) and adsorption capacity (31.02 mg/g) were observed at pH 2. Therefore, the general optimization conditions revealed that the maximum adsorption efficiency of dye was obtained in condition of initial concentration of 20 mg/L, contact time of 1 h and pH of solution equal 2. The adsorption isotherm and kinetic data were evaluated using a series of models. The pseudo-second order kinetic model and Freundlich isotherm model show the best fitting with experimental data with R^2^∼0.999.

## 1. Introduction

Various industries—such as textile, plastic, paper, and food industries—employ over 100,000 types of commercial dyes. Around 10–15% of all dyes used globally, which is equivalent to 280,000 tons, enter the environment through the wastewater discharge from factories [1,2,3]. The consumed dyes are categorized into three groups: non-ionic, cationic, and anionic, which are toxic at low concentrations and have high suitability in water resources. Also, synthetic dyes are categorized as organic dyes which have a complex aromatic molecular structure and are hardly biodegradable [4,5,6]. Therefore, entrance of synthetic dye molecules to water resources has threatened the environment and general health of humans [7]. The high concentration of dyes in wastewaters has negative effects on visibility and with the diminished sunlight penetration into water it impairs photosynthesis, jeopardizing the life of aquaculture, which also interferes with solubility of gases [8,9]. In addition, the chemicals present in dye wastewaters are toxic which cause carcinogenicity, mutagenicity, and teratogenicity in biological species and aquaculture [8]. Therefore, dyes should be removed, for which biological, chemical, and physical methods are available [10]. Their application is evaluated and compared based on critical parameters such as cost, simplicity in design and operation, availability, toxicity of materials, sensitivity, and efficiency [11]. However, most of the mentioned processes are expensive, and thus economical methods should be used.

Recently, hairy nanocellulose, a type of biorenewable cellulose nanoparticles, has been synthesized for methylene blue removal from wastewater by an environmentally friendly and economical procedure, providing an uptake capacity higher than the existing values in the literature [12]. Cellulose nanoparticles were also used for membrane modification, hence, nanocellulose-enabled membranes were tested in dye removal experiments, exhibiting a dye removal efficiency of 70–80% [13]. In addition, modified Cellulose acetate nanofibrous membranes were applied efficiently for removing methylene blue (MB) from water [14]. Furthermore, metal–organic frameworks (MOFs) was tested for anionic and cationic dyes removal by adsorption and a zirconium-metalloporphyrin MOF [15], or a polyoxometalate (POM) composite [2], were used to enhance their adsorption capacity in aqueous solution. Dye adsorption on CO_2_-activated chitosan was studied recently, providing a simple, economical, and quick operation [16].

Adsorption is widely used in water and wastewater treatment [17,18,19,20,21,22,23,24,25,26,27,28], which is one of the most effective technology [29,30,31,32,33,34]. Adsorption process between liquid and solid phase is strongly affected from temperature [35]. Recently, various nanomaterials have been synthesized as absorbents to use for contaminant separation from liquid phase [36]. Nanoscale materials, due to their unique properties—such as large surface area, large number of reactive sites, small size, and high capacity of recovery—have shown excellent performance in solving of many problems related to water quality [7,37].

Acid dyes are commonly used to dye some fibers such as synthetic polyamide, i.e., nylon; natural proteins, i.e., wool and silk; acrylics; and also a blend of these fibers [38]. These acid dyes owe their name to the fact that they are applied in acidic or neutral conditions. Although color index definition, in the category of acid dyes, considers also metal complex dyes and Cr-complex dyes, this article is limited to the classic acid dyes, those applied on wool, silk, and nylon. Commercially existing dyes are mainly acid based azo, anthraquinone or triphenyl methane dyes, with the most popular chromophore being the azo group. Despite the fact that there are several other acid dyes—like xanthane, azine, nitro, quinoline, indigoid, and carbolan—their commercial use is limited.

Regarding all these, this type of dye is more commonly applied in industrial wastewater, with the azo dyes covering the 70% of total dyes worldwide [39]. Their decomposition in the natural environment, becomes difficult due to the presence of various functional groups that have toxic, mutagenic, and carcinogenic effects [40] and therefore must be removed from the effluent before being discharged into natural water systems [41,42,43,44,45].

Hexagonal barium ferrites composites have been previously applied owing to the expectation of high remaining magnetization and high energy product by using micromagnetic calculations, high-density magnetic recording, microwave devices, and magnetofluid medicine [46]. Roy et al. (2009) examined the use of BaFe_12_O_19_/Ni_0.8_Zn_0.2_Fe_2_O_4_ composite powders finding out the effect of temperature coercivity [47]. Later, Radmanesh et al. (2012) studied the use of SrFe_12_O_19_/Ni_0.7_Zn_0.3_Fe_2_O_4_ composite powders, providing the alteration of the reduced remanence (M_r_/M_s_) with the increase of weight fraction of the soft phase and explaining the exchange and bipolar interactions in coordinating the magnetic properties of nanocomposites [48]. These studies examine only the effect of different factors on magnetic properties. Beside the fact that there are several examples regarding the use of composite ferrites as adsorbents [49], the synthesized composite nanoferrite (barium/cobalt) has not been examined so far as an adsorbent.

In the current study, BaFe_12_O_19_/CoFe_2_O_4_@polyethylene glycol nanocomposites (abbreviated as BFO/CFO@PEG) were provided with sol–gel method as nanoadsorbents for the removal of a model dye pollutant, Acid Blue 92 (abbreviated as AB92), from aqueous solutions. The application of this nanocomposite to remove AB92 from effluents is novel and is not tested until now (based on literature screening). Synthetic dyes are one of the most dangerous type of dyes that found in industrial wastewater [50,51,52,53,54,55,56,57,58] and should to be reduced to allowable amount before being discharged into the environment [59]. Then, the effect of changing the parameters such as pH, dye concentration, contact time, and dosage of adsorbent on the adsorption equilibrium and dye removal by BFO/CFO nanocomposites were investigated. Finally, the adsorption kinetics and isotherm models have also been studied.

## 2. Material and Methods

### 2.1. Materials

Cobalt nitrate (Co(NO_3_)_2_6H_2_O) (99%, supplied by Merck, Germany) barium nitrate(Ba(NO_3_)_3_-9H_2_O) (99%, supplied by Merck), iron nitrate (Fe(NO_3_)_3_‧9H_2_O) (99%, supplied by Merck), poly(ethylene glycol) (Sigma-Aldrich (Berlin, Germany)), citric acid (C_6_H_8_O_7_), and sodium hydroxide (NaOH) were applied as received. In all experiment steps, deionized water was used. In this study, in order to prepare the experimental solution containing dye as contaminant, AB92 (anazolene sodium, C_26_H_16_N3Na3o10S3) was purchased from AlvanSabet Corporation (Hamadan, Iran).

**Figure 1:**
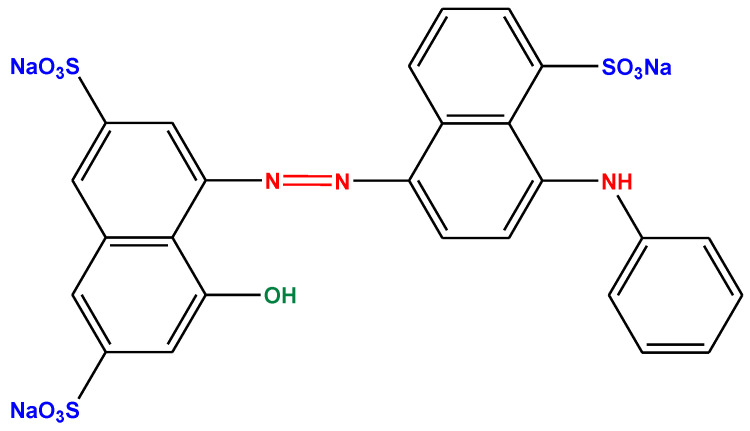
Structure of AB92.

Figure 1 shows the chemical structure of (AB92) which is known commercial salt consisting of dye and inert product with pure dye content was 40%.

### 2.2. Synthesis

BaFe_12_O_19_/CoFe_2_O_4_@polyethylene glycol nanocomposites (BFO/CFO@PEG nanocomposites) synthesized using via sol-gel method [46].

### 2.3. Characterization Techniques

Scanning electron microscopy (SEM) images were performed at Jeol JSM-6390 LV (Jeol, Tokyo, Japan). The accelerating voltage was 15.00 kV and the scanning was performed in situ on a sample powder. EDAX analysis was done at magnification 10 K and led to the maps of elements and elemental analysis. The FTIR spectra of the samples were taken with a PerkinElmer–FT-IR/NIR spectrometer using KBr disks prepared by mixing 0.5% of finely ground carbon sample in KBr. Pellet made of pure KBr was used as the reference sample for background measurements. The spectra were recorded from 4000 to 400 cm^−1^ at a resolution of 4 cm^–1^. The spectra presented are baseline corrected and converted to the transmittance mode. Also, thermal analysis was carried out using a TA Instrument thermal analyzer (SDT). The instrument had the following settings: (i) heating rate of 10 K/min, and (ii) flow rate of nitrogen atmosphere equal to 100 mL/min. Approximately 25 mg of sample was used for each measurement. Moreover, X-ray powder diffraction (XRD) patterns were recorded on a D8 Advance X’Pert X-ray diffractometer (Bruker, Massachusetts, USA) with a CuKα radiation for crystalline phase identification. The sample was scanned from 20 to 80°. The magnetic behavior of the samples was studied using vibrating sample magnetometry (VSM, Kavir Precise Magnetic, Karshan, Iran). Zeta potential measurements were performed using a zeta sizer (Nano Zs, Nano series Malvern instruments, Malvern Panalytical, Cambridge, UK). Measurements were taken in water and PBS. Zeta potential measurements were done three times for each sample at 30 electrode cycles.

### 2.4. Adsorption Experiments

In this study, batch condition was used for all adsorption experiments. In each step of adsorption process, the initial and final concentrations of dye in experimental solutions were measured by UV–vis spectrophotometer (Shimadzu Model, CE-1021, UK) at 571 nm (λ_max_). Also, the MIT65 pH meter was applied for measure of solution pH.

The dye removal percentage (Equation (1)) and its adsorption capacity (Equation (2)) by the BFO/CFO@PEG nanocomposites were determined as

(1) $$\mathrm{Removal}=(\frac{C_{0}-C_{e}}{C_{0}})\cdot 100\%$$

(2) $$Q_{e}=\frac{(C_{0}-C_{e})V}{m}$$

where C_e_ and C_0_ are the equilibrium and initial concentrations of AB92 (mg/L), respectively, V is the volume of solution (L) and m is mass of BFO/CFO@PEG nanocomposites (g) [60].

All adsorption experiments were conducted in triplicate and finally the mean value are reported [61]. For preparation of the stock dye aqueous solution of 1000 mg/L, 1 g of dye was added to a 1 L volumetric flask.

For the determination of dye solution and its mechanism in each particular pH, 0.1 g/L synthesized BFO/CFO@PEG nanocomposites were mixed with 50 mL of dye solution (80 mg/L). Then the pH value of solution was adjusted in range of 2 to 12 by using HNO_3_ (0.01 mol/L) and NaOH (0.01 mol/L). The suspensions were shaken for 40 min at 25 °C (water bath (Julabo SW-21C)). The agitation rate was adjusted at 150 rpm, because over this value the adsorption is not increased, based on results of preliminary tests. As in this study, optimum pH was found in pH = 2, this value was selected for next series of experiments.

In order to investigate the effect of initial dye concentration on its adsorption process by synthesized adsorbent, 1 g BFO/CFO@PEG nanocomposites was mixed with 50 mL of dye solution in different initial concentration (20–100 mg/L). After that the suspensions were agitation with speed of 150 rpm for 40 min at 25 °C.

In equilibrium experiment, the effect of initial concentration was investigated by adding 0.1 g/L of BFO/CFO@PEG nanocomposites with 50 mL solution contain different concentrations of dye (20–100 mg/L). Similar to the pervious step, pH = 2, contact time = 45 min, agitation rate = 150 rpm, and temperature of experiment = 25 °C were selected.

In this study, experiments related to contact time (5, 15, 30, 45, 60, 90, 120, 150, and 180 min) in adsorption kinetic studies were performed by adding 0.1 g/L of synthesized adsorbent with 50 mL of dye solution. Then samples were shaken with agitation rate of 150 rpm, at pH = 2 and temperature of 25 °C.

The experiments of dosage adsorbent effect on dye adsorption were conducted by adding 0.02–0.1 g/L of BFO/CFO@PEG nanocomposites with 50 mL of dye solution (80 mg/L). The suspension were agitated with speed of 150 rpm for 40 min at pH = 2 and temperature of 25 °C. After adsorption and before analysis, the solutions were filtered by using membrane filter with a pore size of 0.45 µm in order to separate the nanocomposites (adsorbent materials) from the supernatant. The experimental design for adsorption experiments was briefly presented in Table 1.

## 3. Results and Discussion

### 3.1. Characterizations

**Figure 2:**
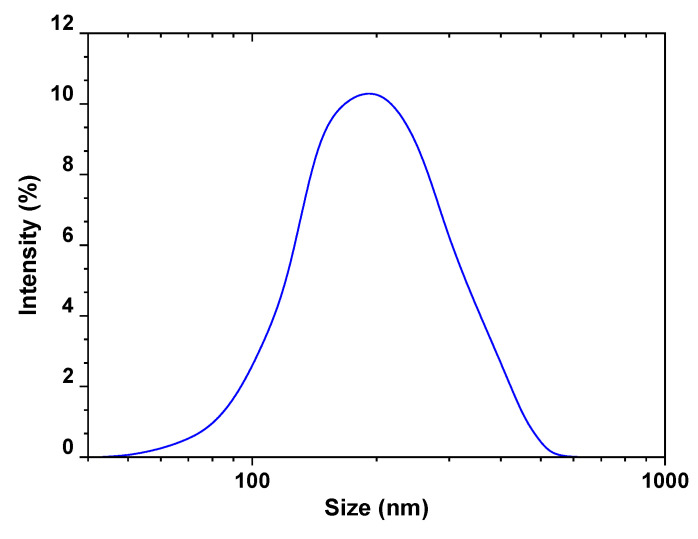
Size distribution of the the prepared nanocomposite.

**Figure 3:**
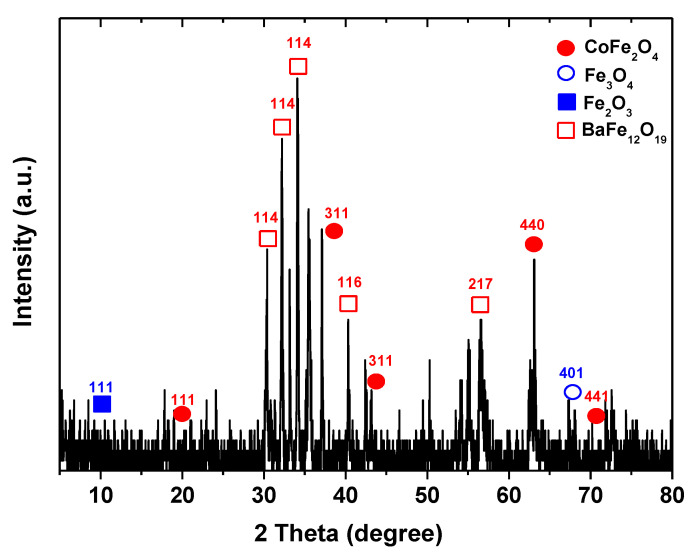
XRD pattern of BFO/CFO@PEG nanocomposites.

Figure 2 presents the size distribution of the prepared BFO/CFO@PEG (peak at 190 nm), while Figure 3 shows the XRD pattern of BFO/CFO@PEG nanocomposites. The presence of hexagonal BaFe_12_O_19_ (JCPDS# 00–043–0002) and cubic spinel CoFe_2_O_4_ (JCPDS# 00–022–1086) is clear. The mean of crystallite size D (nm) of nanocomposite was determined as 75–87 nm, according to (311) plane reflection from the XRD pattern using Debye–Scherrer equation (Equation (3)).

(3) $$D=\frac{K_{s}\cdot \lambda }{B\cdot \cos\theta }$$

where K_s_ is a constant, λ(nm) is wavelength, B is the peak width of half-maximum (rad), and θ is the diffraction angle. Note that for CuKa the relative constant values are K_s_ = 0.9 and λ = 0.15405 nm.

**Figure 4:**
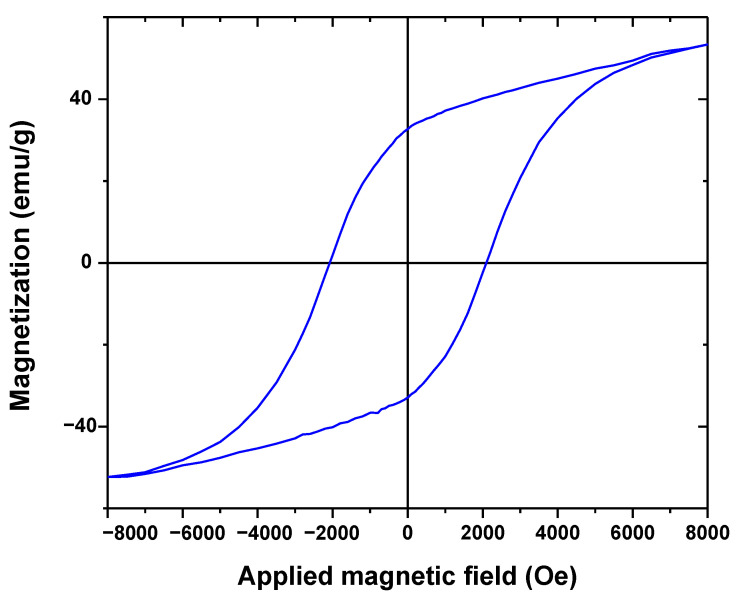
VSM plot of BFO/CFO@PEG nanocomposites.

VSM technique was applied to investigate the magnetic behavior of BFO/CFO@PEG nanocomposite, as illustrated in Figure 4. The ferromagnetism property of nanocomposite, confirmed by the magnetic hysteresis loop. As depicted from Figure 4, there is a VSM plot exhibited a value of 53 emu/g regarding the saturation magnetization of BFO/CFO@PEG. This results also confirmed from the relative literature, concerning the pure magnetite colloidal nanocrystals (36.941 emu/g) [62]. Noticeably, by using an external magnetic field, it can be observed that the nano-adsorbent can be separated relatively easily from the supernatant after adsorption process because of its magnetized properties.

**Figure 5:**
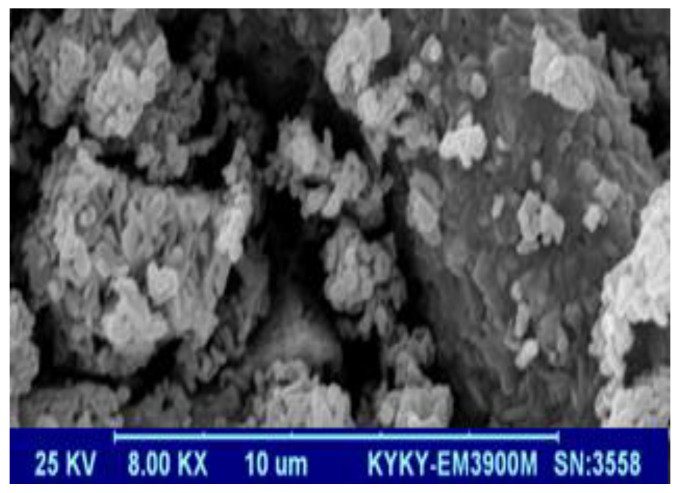
SEM image of BFO/CFO@PEG nanocomposites.

Figure 5 presents the SEM image of BFO/CFO@PEG nanocomposites. As it can be shown, there are both cubic and hexagonal particles in the nanocomposite, which is in agreement with mineral composition determination by XRD analysis. However, the surface morphology is not smooth with some channels and abnormal cavities which can be attributed to the synthesis followed.

**Figure 6:**
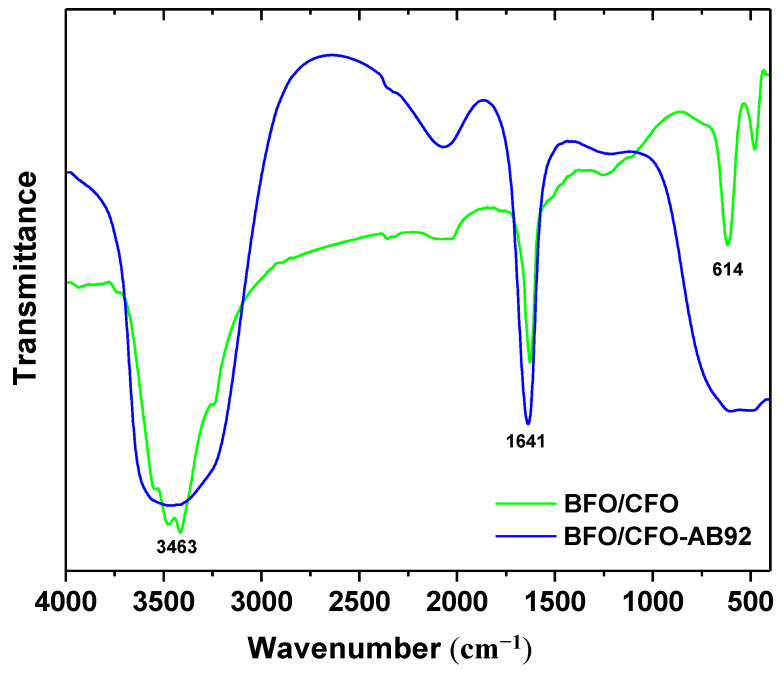
FTIR spectra of BFO/CFO@PEG nanocomposites before and after the adsorption experiment.

The FTIR spectra of BFO/CFO@PEG nanocomposites before and after AB92 adsorption are given in Figure 6. The broad peak at around 3463 cm^−1^ was attributable to the H–stretching, and the band at around 1641 cm^−1^ could be ascribe to H–OH bending vibration. These two peaks of nanocomposite before AB92 adsorption could be attributed to adsorbed water. The changes of the two peaks of nanocomposite after AB92 adsorption resulted from the presence of AB92 on the surface of nanocomposite. The fact that BFO/CFO@PEG was coated effectively to the magnetic Fe_3_O_4_ nanoparticles through electrostatic interaction, was proved by the appearance of a peak around 614 cm^−1^, indicative of the existence of Fe–O stretching. After adsorption test it was observed that the peaks intensity increased significantly, which confirmed the adsorption of AB92 onto nanocomposite.

**Figure 7:**
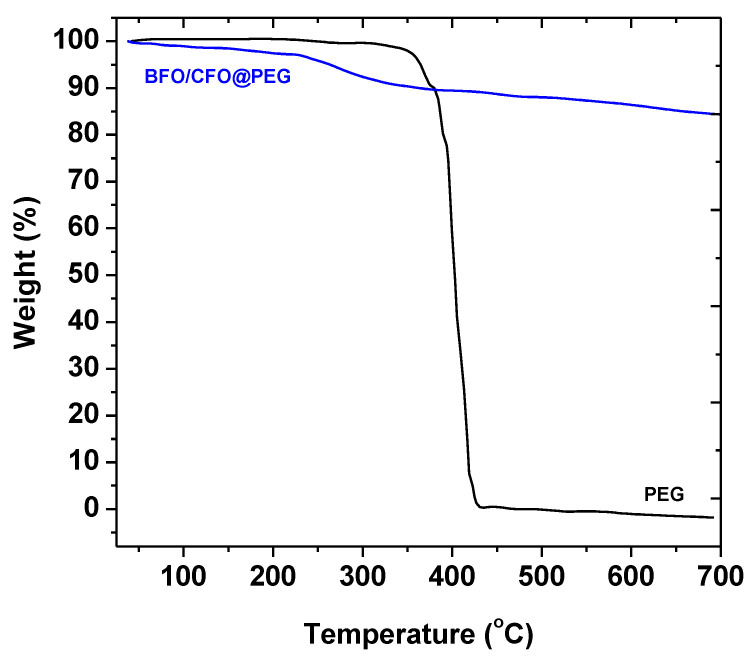
Thermal analysis of PEG and BFO/CFO@PEG.

Thermal stability of BFO/CFO and PEG has been analyzed using TGA (Figure 7). TGA was performed in the temperature range of 25–700 °C to further confirm the existence of PEG on the surface of BFO/CFO nanoparticles and quantify the proportion of organic and inorganic phases. Pure PEG has started to combust at ∼330 °C and completely combusted at ~430 °C. Evidently, the combustion of PEG coated BFO/CFO nanoparticles starts earlier than the decomposition of pure PEG and continues up to 700 °C. The earlier part of combustion curve is due to the evaporation of organic solvents and water. The second part (between 220 and 430 °C) corresponds to decomposition of PEG and BFO/CFO composites into oxides. The temperature at which the decomposition of PEG coated BFO/CFO nanoparticles starts is lower than that of PEG alone, which indicates the catalytic effect of BFO/CFO nanoparticles on the degradation of the PEG. Nanocomposites show a major weight loss of ~20% over the temperature range of 25–700 °C due to the decomposition and combustion of PEG. This implies that nanocomposites have approximately 80% inorganic phase as BFO/CFO nanoparticles.

**Figure 8:**
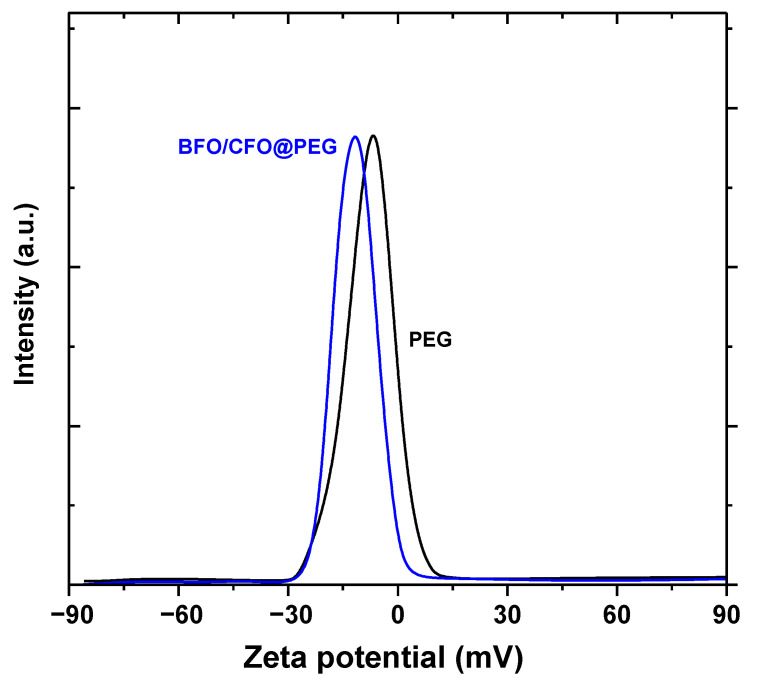
Zeta potential distribution of BFO/CFO and BFO/CFO@PEG nanoparticles (distilled water as dispersant).

The colloidal stability of the BFO/CFO and BFO/CFO@PEG materials was evaluated by zeta potential measurements. The zeta potential distribution of uncoated and coated sample using distilled water as a dispersant is shown in Figure 8. The zeta potential value in distilled water observed for the BFO/CFO@PEG (−12.28 mV) is higher than the uncoated sample BFO/CFO (−9.44 mV). This result shows that there is lesser aggregation of the BFO/CFO@PEG nanocomposite in water compared to the BFO/CFO. High charge differences (> ±10 mV) lead to greater interparticle repulsion [63] hence, there is enhancement of colloidal stability with increasing zeta potential values. Solubility in aqueous medium like water increases due to the hydrophilic ethylene glycol repeats in the PEG coating.

The results show that the BFO/CFO@PEG nanocomposites are relatively colloidally stable both in aqueous and physiological environments. These results imply that the BFO/CFO@PEG nanocomposites could maintain their dispersion stability and heating capacity in various physiological environments and thus have great potentials to be used in many applications.

### 3.2. Adsorption Evaluation

#### 3.2.1. pH Effect

**Figure 9:**
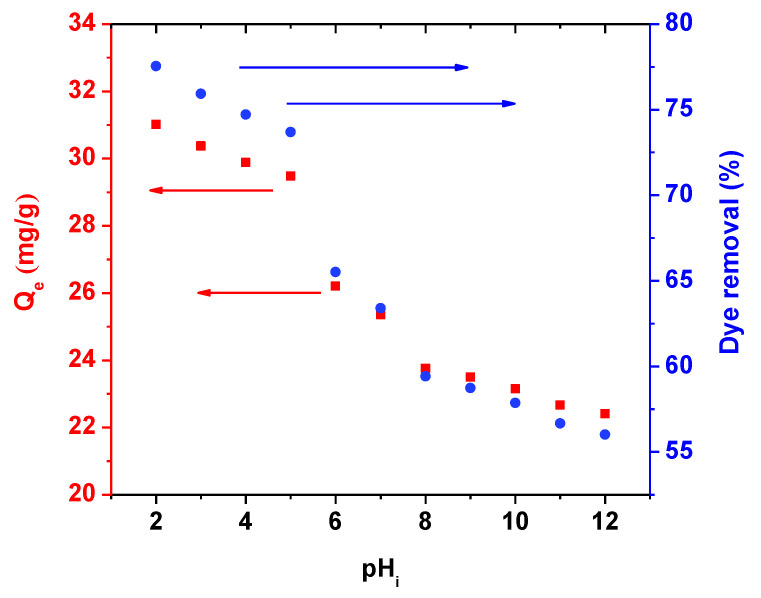
Effect of initial pH of solution on AB92 adsorption by BFO/CFO nanocomposites.

Figure 9 illustrates the effect of initial pH of solutions on the adsorption of AB92 by BFO/CFO@PEG nanocomposite. The adsorption efficiency continuously increased with the reduction of initial solution pH from 2 to 12. The maximum removal efficiency (77.54%) and adsorption capacity (31.02 mg/g) were observed at pH 2. The above finding can be easily explained taking into consideration that pH can change the superficial chemistry of nanoparticles. Therefore, under acidic conditions, H^+^ ions accumulated on the surface of BFO/CFO@PEG nanocomposites [64]. These ions caused momentary neutralization of the negative charge of the nanoparticle surface by developing a buffer effect [65]. Meanwhile, the reduction of pH also provided the conditions for bonding negative sulfonate groups of AB92 molecules [66]. The reduction of surface negative charge posed a positive effect on adsorption of AB92 onto BFO/CFO@PEG nanocomposites.

#### 3.2.2. Effect of Adsorbent Dosage

**Figure 10:**
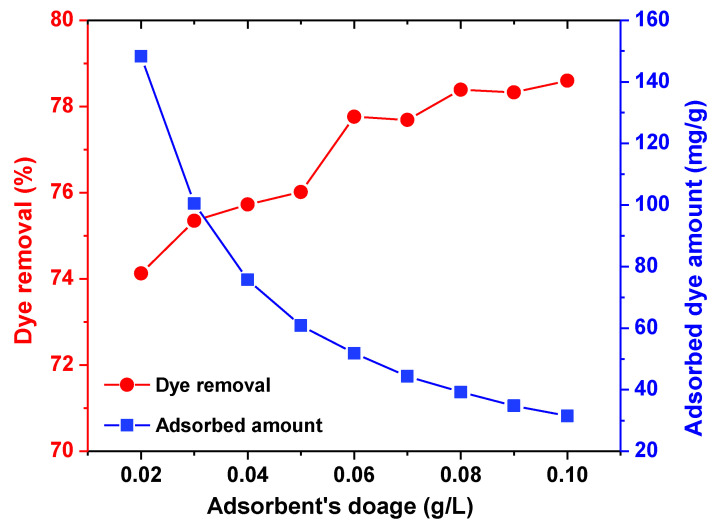
Effect of adsorbent dosage on AB92 adsorption by BFO/CFO nanocomposites.

Figure 10 shows the effect of adsorbent dosage on the adsorption of AB92 by BFO/CFO@PEG nanocomposites. The results indicated that with the increase of the adsorbent dosage from 0.02 to 0.1 g/L, the removal efficiency increased from 74.1 to 78.6%, and the adsorbed amount decreased from 148.25 to 31.44 mg/g. At the adsorbent dosage of 0.06 g/L, relatively high removal efficiency (77.76%) and adsorption capacity (51.84 mg/g) were achieved. The increase of adsorbent’s dosage means the increased number of active sites and the available surface centers of nanocomposite, causing the expected improvement of removal efficiency [61,67]. Meanwhile, the adsorbed amount of nanocomposite reduces by increasing the dosage, as some available sites on the absorbent surface had not been saturated and the adsorbate to absorbent ratio declined [68,69]. As the reactivity of nanocomposites are high, with increasing in the adsorbent dosage, adsorbent particle may be aggregated. Therefore their surface area and available adsorption sites on synthesized adsorbent will be reduced and finally AB92 adsorption decrease.

#### 3.2.3. Isotherms and Kinetics

**Figure 11:**
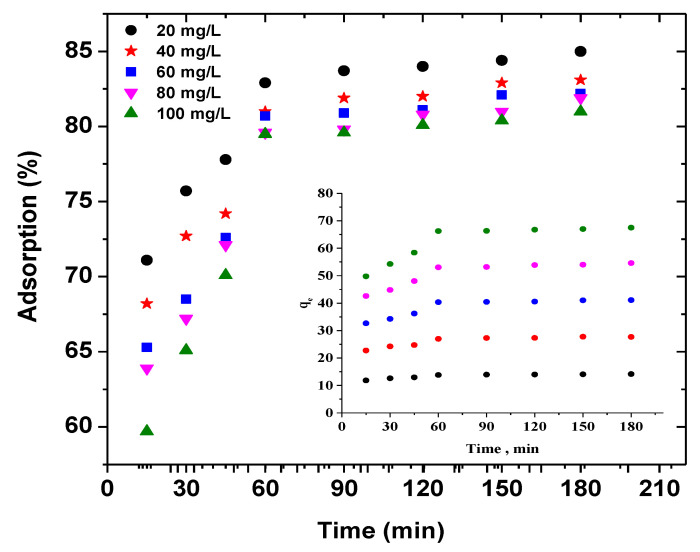
Effect of contact time and initial dye concentration on the adsorption of AB92 by BFO/CFO@PEG nanocomposite.

Figure 11 shows the effectiveness of the adsorption of AB92 by BFO/CFO@PEG nanocomposite, taking into account the effect of contact time and initial dye concentration. As depicted, when the initial concentration was low, a rapid adsorption of the dye molecules on the surface of the nanocomposite was observed, while with the increasing of concentration a saturation was detected on the surface, thereby reducing the removal efficiency [70]. Figure 9 also indicated the adsorption tended to be equilibrium after 60 min, and after that, the elevation of removal efficiency was limited.

#### 3.2.4. Isotherm Models

The isotherm of dye adsorption on BFO/CFO@PEG nanocomposite was investigated with different available models such as Langmuir, Freundlich, Temkin, and Dubinin-Radushkevich. Also the goodness of applied models was investigated by coefficient of determination (R^2^). When the adsorption sites of the adsorbents are uniform and the adsorbent surface is homogenous, there will be equal energy and enthalpy for all adsorbate molecules in the adsorption process. The linear of Langmuir model is given below [71].

(4) $$\frac{1}{q_{e}}=\frac{1}{q_{m}}+(\frac{1}{q_{m}K_{L}})\frac{1}{C_{e}}$$

where q_e_ (mg/g) is the amount of dye adsorbed per unit weight of BFO/CFO@PEG nanocomosite at equilibrium time, C_e_ (mg/L) is the equilibrium concentration of dye in solution, q_m_ (mg/g) is the maximum adsorption capacity of BFO/CFO@PEG nanocomosite, and K_L_ (L/mg) is the Langmuir equilibrium constant.

However, if the nanoparticle surface is heterogenous, Freundlich relation obtained by measuring the amount of adsorbed material at different pressures offers a better description of data [72].

(5) $$\log\left(q_{e}\right)=\frac{1}{n}\log\left(C_{e}\right)+\log\left(K_{F}\right)$$

where K_F_ (mg/g). (L/g)^n^ is the constant of equilibrium adsorption of Freundlich and n is the heterogeneity factor.

Temkin model is obtained based on indirect interaction of the adsorbent and adsorbate [73]

(6) $$q_{e}=B\ln(K_{T})+B\ln(C_{e})$$

where B is related to the heat of adsorption (L/g), and K_T_ is dimensionless Temkin isotherm constant.

Type of dye adsorption on the BFO/CFO@PEG nanocomosite in nature (physical or chemical) was determined by using Dubinin-Radushkevich isotherm model [74].

(7) $$\log\left(q_{e}\right)=\ln\left(q_{m}\right)-\beta \cdot \varepsilon^{2}$$

where q_e_ (mg/g) is the amount of dye adsorbed per unit weight of BFO/CFO@PEG nanocomosite at equilibrium time, q_m_ (mg/g) is the maximum adsorption capacity of BFO/CFO@PEG nanocomosite, β (mol^2^/J^2^) is a coefficient of mean adsorption energy.

The parameters of isotherm models are presented in Table 2.

#### 3.2.5. Kinetic Models

In this study, different kinetic models such as pseudo-first-order (Equation(8)), pseudo-second-order (Equation (9)), intraparticle diffusion (Equation (10)), and Ritchie (Equation (11)) were used to describe the dye adsorption kinetic data [75]. The linear forms of first-order-model and pseudo-second-order are presented as

(8) $$\log\left(q_{e}-q_{t}\right)=\log\left(q_{e}\right)-t\frac{k_{1}}{2.303}$$

(9) $$\frac{t}{q_{t}}=\frac{1}{k_{2}q_{e}{}^{2}}+t\frac{1}{q_{e}}$$

where q_t_ (mg/g) is the amount of dye adsorbed on BFO/CFO@PEG nanocomposite at time t, q_e_ (mg/g) is the amount of dye adsorbed on BFO/CFO@PEG nanocomposite at equilibrium time, k_1_ (1/min) is the rate constant of the pseudo-first-order, and k_2_ (g/mg·min) is the rate constant of pseudo-second-order model.

Chemisorption of pollutant on the adsorbent is responsible for the pseudo-second- order model.

The intraparticle diffusion kinetic model is used for investigating the mechanism of diffusion of dye in porous nanoparticles and with transferring dye molecules from the soluble phase into the pores as the stage for determining and controlling the adsorption rate.

(10) $$q_{t}=k_{p}t^{1/2}+C$$

where k_p_ (mg/g. min^0.5^) is the rate constant of intraparticle diffusion and C (mg/g) is the intercept which shows surface adsorption or effect of boundary layer.

The Ritchie kinetic model is presented as

(11) $$\frac{1}{q_{t}}=\frac{1}{t}\cdot \left(\frac{1}{q_{e}k_{r}}\right)+\frac{1}{q_{e}}$$

where q_t_ and q_e_ (mg/g) are the amount of dye adsorbed on BFO/CFO@PEG nanocomposite at time t and equilibrium time, respectively and k_r_ is the rate constant (1/min).

**Figure 12:**
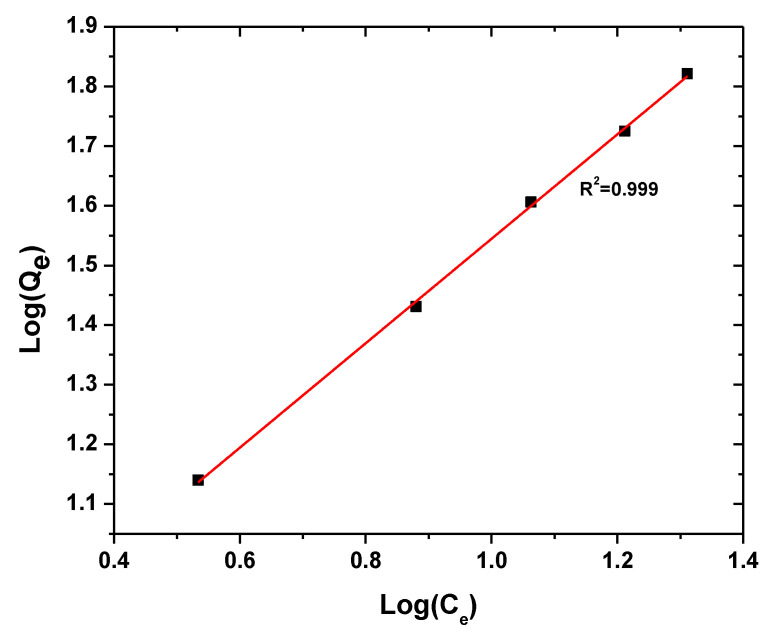
Freundlich isotherm plot of dye adsorption on BFO/CFO@PEG nanocomposites. Experimental condition: initial dye concentration 20–100 mg/L, initial solution pH 2, adsorbent dosage 0.06 g/L, and contact time 40 min.

**Figure 13:**
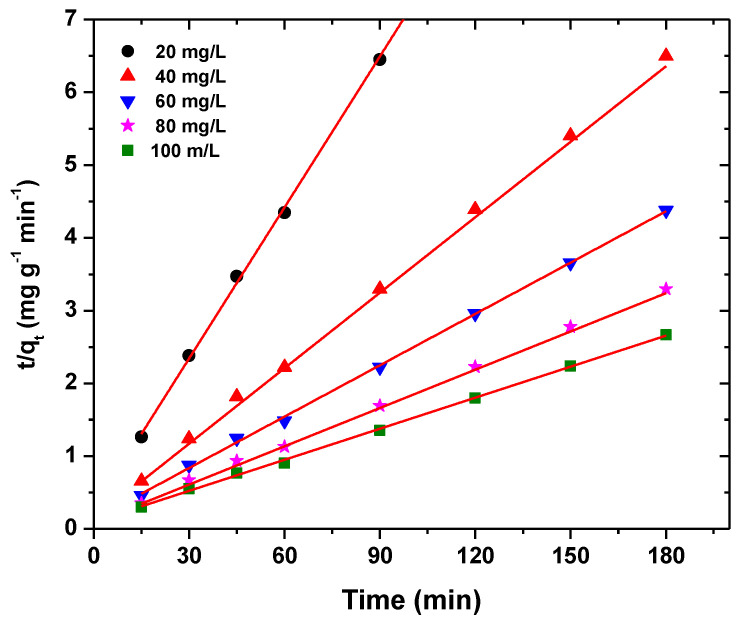
Pseudo-second-order model for dye adsorption by BFO/CFO@PEG nanocomposites. Experimental condition: initial dye concentration 20–100 mg/L, initial solution pH 2, adsorbent dosage 0.06 g/L, and contact time 40 min.

The parameters of all the kinetic models are presented in Table 3. In terms of *R*^2^ values, the pseudo-second-order kinetic and Freundlich isotherm models showed the most satisfactory fits. Figure 12 and Figure 13 illustrate the plots of the Freundlich isotherm and pseudo-second-order kinetic models, respectively.

### 3.3. Comparisons

A brief comparison regarding the application of various nanocomposites for dye removal is very important to check the performance of our system (adsorbent/adsorbate). Some recent examples are given in following.

Zhou et al. (2011) [76] studied effect of application ethylenediamine-modified magnetic chitosan nanoparticles (EMCN) to remove Acid orange 7 and Acid orange 10 from aqueous solution. The result of their research showed that Langmuir isotherm model was the best applicable model and maximum adsorption capacity of applied adsorbent for adsorption Acid orange 7 and Acid orange 10 at temperature of 298 K was obtained to be 3.47 and 2.25 mmol/g, respectively. Zhang and Kong [77] described the adsorption of orange dyes from aqueous solution by synthesized magnetic Fe_3_O_4_/C core–shell nanoparticles. The maximum adsorption capacity of synthesized adsorbent for Cr and MB in studied range was 11.22 and 44.38 mg/g respectively. Xie et al. [78] in order to adsorption of different dyes such as neutral red (NR), methylene blue (MB), and methyl orange (MO) by magnetic HNT-Fe_3_o_4_ as adsorbent were carried a research. Magnetic HNT-Fe_3_o_4_ shows the best performance for adsorption of MB (18.44 mg/g). Also adsorption capacity of mentioned adsorbent for adsorption of NR and MO was 13.62 and 0.65 mg/g respectively. Inbaraj and Chen (2011) [79], investigated the application of magnetite nanoparticles coated with an anionic biopolymer for dye adsorption. Results of their study present that Redlich-Peterson and Langmuir equations were the best isotherm models. Also, the kinetic data was predicted with pseudo-second-order model as well. The maximum adsorption capacity of synthesized adsorbent was obtained 78.67 mg/g. Debrassi et al. [80,81], were synthesized the magnetic nanoparticles with iron oxides and N-benzyl-O-carboxymethylchitosan by incorporation method. Based on obtained results, the Langmuir–Freundlich model showed the best fitness with experimental data. Maximum adsorption capacity of synthesized adsorbent for MB, CV, and MG adsorption was determined 223.58, 248.42, and 144.79 mg/g. Singh et al. (2011) [82], investigated the efficiency a novel magnetic carbon-iron oxide nanocomposite (MCIONC) to remove of crystal violet dye from aqueous solution. Based on the results of Langmuir model, maximum adsorption of CV under optimum condition was 113.31 mg/g. while in batch experiment, maximum adsorption capacity of adsorbent determined 111.80 mg/g. Yao et al. (2012) [83], explained the dye adsorption (MB and CR) by magnetic Fe_3_O_4_@graphene composite from water. The results their study showed each unit of adsorbent could be to remove 45.27 and 33.66 g MB and CR, respectively. Mahmoodi (2013) [84] applied the magnetic ferrite nanoparticles (MFN)-alginate composite for dyes removal (Basic Blue 9 (BB9), Basic Blue 41 (BB41), and Basic Red 18 (BR18)).obtained results present that maximum adsorption capacity of (MFN)-alginate composite for adsorption of BB9, BB41, and BR18 was 106 mg/g, 25 mg/g, and 56 mg/g respectively. Fan et al. (2012) [85] used magnetic chitosan and grapheme oxide composite for methylene blue dye removal from water. The results their research showed under condition of initial concentration of 10 mg/g and pH 10, maximum adsorption obtained 179.6 mg/g.

### 3.4. Perspectives/Limitations

Adsorption is a very promising technique and some important advantages are be listed below [86]:

- High ability to remove various organic and inorganic pollutants.
- Existence of a wide range of new adsorbent with high adsorption capacity.
- Use of cheap absorbents.
- Fast and simple procedure of removing pollutants.
- The ability to reuse some used adsorbent.
- The cost of the initial investment is cheap.

On the other hand, there are major limitation of the process as [86]:

- Dependence of performance on the type of used adsorbent.
- Low capacity of some adsorbent to remove various pollutants.
- Rapid saturation of some adsorbents and reduction of their adsorption capacity.
- Non-selective pollutant for removing in a binary solution.
- Problems related to desorption of the used adsorbent after the adsorption process.
- Dependence of the adsorption process to various parameters such as temperature, time, and pH.

Nanoparticles that used as adsorbent due to small size, their recovery is very difficult and expensive. Generally, sedimentation and filtration techniques are applied for their separating from aqueous solutions. Uses of the mentioned method have some challenges that reduce the pollution adsorption efficiency. for example blockage of filters due to small dimension of nanoparticles, secondary pollution due to spreading of nanoparticles, and reducing of adsorption efficiency due to the tendency of nanoparticles to agglomeration [87]. Therefore, to solve the mentioned problems related to nanoparticles, the solutions have been used. One of the solutions is combination of nanoparticles with materials of larger dimension (active carbon and nanofibers) or combination with magnetic material so that their use and reused become easy, fast, simple, and inexpensive [88].

Moreover, experimental research of dye removal from industrial wastewater by nanoadsorbents at laboratory scale consider only one type contaminant while, in fact, a sample of industrial effluent contains a variety of dyes and contaminants.

Another challenge in that using nanoparticles to remove dye is a costly procedure due to the effort required for preparing nanoparticles as adsorbents. For example TIo2 nanoparticles and CNTs are the materials that use a lot for removing of different pollutant such as dye, while its preparation is complex, toxic and expensive. More importantly, the use of nanoparticles may cause other environmental problems due to its small size and high dispersal ability in water, soil, etc. Hence, it is necessary to investigate the toxicity and performance of nanoparticles before any applications.

## 4. Conclusions

BFO/CFO@PEG nanocomposite was demonstrated to be an efficient adsorbent for adsorption of AB92 from the aqueous solution. The adsorption process was dependent on different conditional parameters such as contact time, solution pH, initial dye concentration, and adsorbent. The effects of conditional parameters including pH (2–12), initial concentration of dye (20–100 mg/L), adsorbent dosage (0.02–0.1 g/L), and contact time (0–180 min) on the adsorption of dye were investigated and then optimized. A high removal efficiency of 88% was achieved under the optimal condition of adsorbent dosage of 0.06 g/L, contact time of 60 min, initial dye concentration of 20 mg/L, and solution pH 2. The pseudo-second-order kinetic model and Freundlich isotherm model properly describe the experimental data of this research in adsorption process.

## Figures and Tables

**Table 1:** Experimental conditions AB92 adsorption from aqueous solutions by BFO/CFO@PEG nanocomposites.

Experiment	pH_i_	T (°C)	C_0_ (mg/L)	N (rpm)	t (min)	m/V (g/L)
Effect of pH	2–12	25	80	150	40	0.1
Effect of dosage	2	25	80	150	40	0.02–0.10
Effect of contact time	2	52	80	150	0–180	0.06
Effect of initial dye concentration	2	25	20–100	150	180	0.1

**Table 2:** Equilibrium parameters of fitting to various isotherm models.

Isotherm Model	Parameters	
Langmuir	K_L_ (L/ mg)	0.02
	q_m_ (mg/g)	215.08
	R^2^	0.998
Freundlich	K_F_(mg/g)	4.64
	n	0.14
	R^2^	0.999
Temkin	K_T_	5.046
	B	0.032
	R^2^	0.994
Dubinin–Radushkevich	β	0.0142
	q_m_ (mg/g)	3.142
	R^2^	0.959

**Table 3:** Kinetic parameters of fitting to various isotherm models.

Kinetic Model	Parameters	
Pseudo-first-order	k_1_ (1/min)	1.001
	q_e_ (mg/g)	1.024
	R^2^	0.921
Pseudo-second-order	K_2_ (g/mg.min)	0.0177
	q_e_ (mg/g)	14.471
	R^2^	0.9998
Intra-particle-diffusion	K (mg/g.min)	0.23
	C (mg/g)	11.409
	R^2^	0.83
Ritchie	k_r_	0.296
	q_e_ (mg/g)	14.347
	R^2^	0.936

## Data Availability

The data presented in this study are available upon request from the corresponding author.
